# Comparison of treatment strategies and survival of early-onset gastric cancer: a population-based study

**DOI:** 10.1038/s41598-022-10156-5

**Published:** 2022-04-15

**Authors:** Chunmei Zhang, Ruiyi Tang, Hanlong Zhu, Xianxiu Ge, Yue Wang, Xue Wang, Lin Miao

**Affiliations:** 1 Medical Centre for Digestive Diseases, the Second Affiliated Hospital of Nanjing Medical University, 121 Jiangjiayuan Road, Nanjing, 210011 Jiangsu China; 2 Department of Gastroenterology and Hepatology, Jinling Hospital, Medical School of Nanjing University, Nanjing, 210002 Jiangsu China

**Keywords:** Cancer, Gastroenterology, Oncology

## Abstract

Treatments for early-onset gastric cancer (EOGC) patients are rarely included in clinical trials, resulting in an unclear impact on survival. This study aimed to investigate the treatment patterns of EOGC patients and their impact on survival. Based on the Surveillance, Epidemiology, and End Results database, we conducted a retrospective analysis of 1639 EOGC patients (< 50 years) diagnosed between 2010 and 2018. Patients with larger tumours, distant metastasis, and AJCC TNM stage in IV were prone to receive nonsurgical treatment. Patients treated with surgery alone had a better prognosis than those receiving SROC or SCRT or nonsurgical treatment. However, analyses stratified by histological type, tumour size and TNM stage showed that patients did not benefit more from SROC and SCRT than from surgery alone. Similar results were observed in the stratified Cox regression risk analysis. Patients who received nonsurgical treatment had the highest risk of overall death [hazard ratio (HR) = 2.443, 95% confidence interval (CI) 1.865–3.200, *P* < 0.001]. This study indicated that additional radiotherapy, chemotherapy or chemoradiotherapy did not provide a coordinated survival benefit to EOGC patients.

## Introduction

Gastric cancer (GC) is one of the most common malignant tumours of the digestive system, ranking fifth and fourth for the incidence and mortality rates respectively among all malignant tumours according to the newly released global cancer statistics 2020^1^. Among them, the incidence rate in men is twice as high as that in women^1,2^. It has been reported that GC, mainly caused by environmental factors and genetic alterations^3^, is most prevalent in individuals aged 50–70 years old, but rather rare in the young population (under 50 years old), which is named early-onset gastric cancer (EOGC)^4^. There has been a decrease in the incidence and mortality rate of GC and an increase among young people over the past half century^5–7^. The incidence rate of EOGC fluctuates between 2.7% and 15% based on the different population studies^8–10^.

The appropriate treatment plays an important role in the prognosis of patients with GC, especially in young patients. Previous studies have not clearly distinguished the treatment methods between EOGC patients and ordinary GC patients. Comprehensive surgical resection combined with D2 lymph node dissection is still the main treatment for GC patients^11,12^, and sometimes chemotherapy or radiotherapy is given before or after surgery. A study involving 3083 gastroesophageal junction cancer patients from the National Cancer Center showed that both neoadjuvant chemoradiotherapy and neoadjuvant chemotherapy improved prognosis in patients with comparable survival^13^. However, a Dutch randomized trial showed that preoperative chemotherapy did not improve survival in patients with GC. There was a median overall survival of 18 months in the neoadjuvant chemotherapy group using 5-fluorouracil, doxorubicin and methotrexate group compared with 30 months in the surgery alone group (*P* = 0.170)^14^. Another study also suggested that potential overuse of chemotherapy in EOGC patients and the addition of chemotherapy did not result in a corresponding improvement in survival^15^. Radiotherapy as a single modality has minimal value in patients with unresectable GC^16^. Radiotherapy combined with surgery or chemotherapy could improve survival. In a randomized controlled trial, 370 patients were randomly divided into preoperative radiotherapy group and surgery alone group, and the survival of the former was significantly improved (30% vs. 20%, *P* = 0.0094)^17^. In another trial, compared with patients who underwent surgery alone, those receiving postoperative radiotherapy or chemotherapy did not gain a survival benefit^17^. Another study shown that docetaxel-based chemotherapy regimen was conducive to providing longer survival and lower risk of recurrence and death for patients with signet ring cell carcinoma (SRCC)^18^. However, more studies suggested that gastric cancer with signet-ring cells has long been known to be insensitive to chemotherapy and radiotherapy^19,20^. Accordingly, not every GC patient can benefit from chemotherapy or radiotherapy, and some patients may even be harmed. Previous studies have shown significant differences in biological characteristics and physical fitness between EOGC and ordinary GC patients. Young patients exhibit poor histological tomour differentiation and rapid disease progression^21–23^. Whether these treatments are also suitable for EOGC patients remains unclear, and the optimal treatment strategies in young patients are still controversial.

Therefore, this study aimed to evaluate overall survival (OS) and cancer-specific survival (CSS) in EOGC patients based on four different treatment modalities: surgery only, surgery plus radiotherapy or chemotherapy (SROC), surgery plus chemoradiotherapy (SCRT), and nonsurgical treatment (giving radiotherapy alone or chemotherapy alone or chemoradiotherapy) by analysing the Surveillance, Epidemiology, and End Results (SEER)-registered database.

## Methods

### Data source and population selection

**Figure 1:**
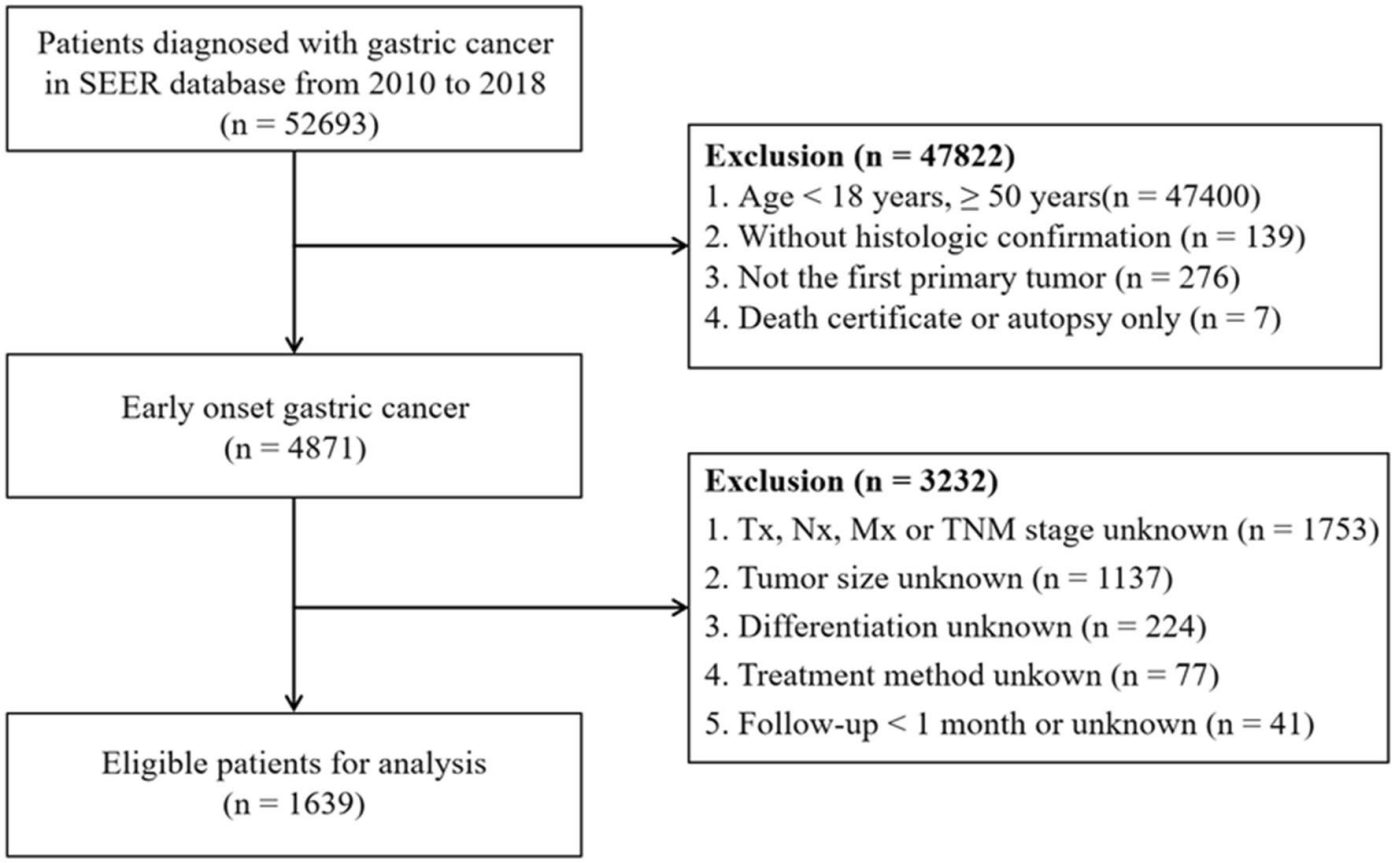
Flow diagram of eligible young patients diagnosed with gastric cancer.

The SEER Program, sponsored by the National Cancer Institute, is one of the largest public databases in the world, providing authoritative information on cancer patients in 19 geographic regions of the United States. For this study, we logged into SEER*Stat software (version 8.3.9.2; NCI, Bethesda, MD, USA) to access the SEER data with the username 15837-Nov2020. The data we selected from the latest dataset, Incidence-SEER Research Plus Data, 18 Registries, Nov 2020 Sub [2000–2018], were released on July 2021. Overall, 52,693 cases were initially identified based on the Behaviour code ICD-O-3 (Malignant) and Site recode ICD-O-3/WHO 2008 (Stomach) from 2010 to 2018. Only those meeting the following criteria were included in this study: (1) age at < 50 years; (2) histopathology confirmed as GC; (3) only one primary tumour; (4) complete information on age, sex, tumour size and the American Joint Committee on Cancer (AJCC) TNM stage; and (5) complete survival time and treatment modality. To avoid immortal time bias, patients with less than 1 month of follow-up were excluded. After a multistep screening process, 1639 EOGC patients were finally enrolled in this study. More screening details are shown in Fig. 1.

### Study variables and subgroups

The variables studied were composed of demographic features (age, sex, race and marital status), tumour-related characteristics (primary tumour site, size, histologic type, T stage, N stage, M stage, TNM stage, liver, lung, brain and bone metastases), treatment methods and follow-up status (survival time, vital status and cause of death) of each patient. In addition, the treatment modalities were divided into four groups taking into account the actual clinical situation.

### Endpoints

The primary and secondary endpoints of our study were OS and CSS, respectively. We defined OS as the period between the date of diagnosis and death for any cause or the last follow-up, while CSS was defined as the duration from diagnosis to the last follow-up or death owing to tumor death.

### Statistical analysis

Descriptive results in each treatment group were expressed as the frequencies and percentages. Multivariate logistic regression analysis was applied to investigate the relevant clinicopathological factors associated with treatment strategies, and the nontreatment group was used as a reference and set to a value of one. The results are presented as the adjusted odds ratios (aOR) with 95% confidence intervals (CI) and *P* values, and an aOR > 1 reflected better odds for selecting treatment. We described the survival status of patients in each treatment group with Kaplan–Meier analysis and log-rank test, layered survival analysis based on histological type, tumour size and AJCC TNM stage. All analyses were performed with the statistical software SPSS 24.0 (IBM Corp, Armonk, NY) for chi-square test, multinomial logistics regression and Cox regression analyses, whereas GraphPad Prism 9 (GraphPad Software, San Diego, CA) was used for Kaplan–Meier survival curves and log-rank test. *P* < 0.05 in two-sided analysis was considered a statistically significant difference.

### Ethical approval

The approval of the institutional review board was not necessary for this study because data from the SEER database are publicly available.

## Results

### Clinical and treatment modality characteristics

In total, 1639 EOGC cases diagnosed at ages < 50 years were selected from the SEER database between 2010 and 2018. The median age at diagnosis was 44 years old (IQR 39–47 years old). More than half of the patients were males (56.4%), married (60.6%), and white (66.1%). There were 1050 (64.1%) patients with gastric adenocarcinoma and 563 (34.4%) patients with SRCC. Moreover, poorly differentiated or undifferentiated EOGC patients accounted for 80.4%. The majority of patients presented with nonmetastatic disease (76.8%), while 380 (23.2%) individuals were positive for distant metastasis, of which 100 (6.1%) had liver metastases, 42 (2.6%) had bone metastases, 29 (1.8%) had lung metastases, 6 (0.4%) had brain metastases, and the rest had other modes of distant metastases. A large proportion of patients exhibited a deeper depth of infiltration in T3/T4 and higher AJCC TNM stage in III/IV. Concerning treatment methods, the majority of patients received surgery, yet only a small percentage accepted conservative treatment (18.2%). Patients in stage I were more willing to undergo surgery alone, while those in stage IV mainly chose conservative treatment. In addition, the II/III stage groups were both more likely to perform SROC or SCRT, and the share of patients was similar in each group. More detailed demographic and cancer characteristics of each treatment subgroup are summarized in Table 1. There was no significant difference in age subgroups (*P* = 0.834), race (*P* = 0.104), or marital status (*P* = 0.280) among the treatment groups, while the remaining variables were significantly different (*P* < 0.05).

**Table 1:** The demographics and characteristics of patients according to treatment received.

Variables	Total	Surgery only	SROC	SCRT	No surgery	*P*-value
	n = 1639 (%)	n = 282 (17.2%)	n = 534 (32.6%)	n = 525 (32.0%)	n = 298 (18.2%)	
**Age groups, years, %**						
≤ 40 years	536 (32.7%)	84 (15.7%)	179 (33.4%)	176 (32.8%)	97 (18.1%)	0.834
41–45 years	509 (31.0%)	88 (17.3%)	169 (33.2%)	165 (32.4%)	87 (17.1%)	
> 45 years	594 (36.2%)	110 (18.5%)	186 (31.3%)	184 (31.0%)	114 (19.2%)	
**Sex, %**						
Male	924 (56.4%)	140 (15.2%)	279 (30.2%)	309 (33.4%)	196 (21.2%)	< 0.001
Female	715 (43.6%)	142 (19.9%)	255 (35.7%)	216 (30.2%)	102 (14.3%)	
**Race, %**						
White	1084 (66.1%)	187 (17.3%)	336 (31.0%)	343 (31.6%)	218 (20.1%)	0.104
Black	218 (13.3%)	38 (17.4%)	73 (33.5%)	77 (35.3%)	30 (13.8%)	
Others^a^	337 (20.6%)	57 (16.9%)	125 (37.1%)	105 (31.2%)	50 (14.8%)	
**Marital status, %**						
Married	993 (60.6%)	158 (15.9%)	339 (34.1%)	326 (32.8%)	170 (17.1%)	0.280
Unmarried	454 (27.7%)	85 (18.7%)	141 (31.1%)	136 (30.0%)	92 (20.3%)	
Others^b^	192 (11.7%)	39 (20.3%)	54 (28.1%)	63 (32.8%)	36 (18.8%)	
**Location, %**						
C16.0/C16.1	488 (29.8%)	43 (8.8%)	92 (18.9%)	197 (40.4%)	156 (32.0%)	< 0.001
C16.2	200 (12.2%)	30 (15.0%)	95 (47.5%)	44 (22.0%)	31 (15.5%)	
C16.3/C16.4	436 (26.6%)	99 (22.7%)	151 (34.6%)	156 (35.8%)	30 (6.9%)	
C16.5/C16.6	253 (15.4%)	48 (19.0%)	93 (36.8%)	71 (28.1%)	41 (16.2%)	
C16.8/C16.9	262 (16.0%)	62 (23.7%)	103 (39.3%)	57 (21.8%)	40 (15.3%)	
**Tumor size, %**						
≤ 3 cm	554 (33.8%)	160 (28.9%)	164 (29.6%)	155 (28.0%)	75 (13.5%)	< 0.001
3.1–5.0 cm	460 (28.0%)	53 (11.5%)	148 (32.2%)	162 (35.2%)	97 (21.1%)	
> 5.0 cm	625 (38.1%)	69 (11.0%)	222 (35.5%)	208 (33.3%)	126 (20.2%)	
**Grade, %**						
I/II	321 (19.6%)	53 (16.5%)	70 (21.8%)	123 (38.3%)	75 (23.4%)	< 0.001
III/IV	1318 (80.4%)	229 (17.4%)	464 (35.2%)	402 (30.5%)	223 (16.9%)	
**Histological type, %**						
Adenocarcinoma	1050 (64.1%)	162 (15.4%)	321 (30.6%)	349 (33.2%)	218 (20.8%)	< 0.001
SRCC	563 (34.4%)	114 (20.2%)	207 (36.8%)	164 (29.1%)	78 (13.9%)	
Others^c^	26 (1.6%)	6 (23.1%)	6 (23.1%)	12 (46.2%)	2 (7.7%)	
**AJCC TNM stage, %**						
I	272 (16.6%)	162 (59.6%)	61 (22.4%)	33 (12.1%)	16 (5.9%)	< 0.001
II	447 (27.3%)	43 (9.6%)	147 (32.9%)	210 (47.0%)	47 (10.5%)	
III	540 (32.9%)	46 (8.5%)	210 (38.9%)	252 (46.7%)	32 (5.9%)	
IV	380 (23.2%)	31 (8.2%)	116 (30.5%)	30 (7.9%)	203 (53.4%)	
**AJCC_T stage, %**						
T1	300 (18.3%)	148 (49.3%)	42 (14.0%)	34 (11.3%)	76 (25.3%)	< 0.001
T2	172 (10.5%)	28 (16.3%)	65 (37.8%)	59 (34.3%)	20 (11.6%)	
T3	626 (38.2%)	44 (7.0%)	196 (31.3%)	282 (45.0%)	104 (16.6%)	
T4	541 (33.0%)	62 (11.5%)	231 (42.7%)	150 (27.7%)	98 (18.1%)	
**AJCC_N stage, %**						
N0	529 (32.3%)	187 (35.3%)	128 (24.2%)	110 (20.8%)	104 (19.7%)	< 0.001
N1	433 (26.4%)	22 (5.1%)	133 (30.7%)	137 (31.6%)	141 (32.6%)	
N2	303 (18.5%)	26 (8.6%)	109 (36.0%)	136 (44.9%)	32 (10.6%)	
N3	374 (22.8%)	47 (12.6%)	164 (43.9%)	142 (38.0%)	21 (5.6%)	
**AJCC_M stage, %**						
M0	1259 (76.8%)	251 (19.9%)	418 (33.2%)	495 (39.3%)	95 (7.5%)	< 0.001
M1	380 (23.2%)	31 (8.2%)	116 (30.5%)	30 (7.9%)	203 (53.4%)	
**Bone metastasis, %**						
Yes	42 (2.6%)	1 (2.4%)	6 (14.3%)	1 (2.4%)	34 (81.0%)	< 0.001
No/unknown	1597 (97.4%)	281 (17.6%)	528 (33.1%)	524 (32.8%)	264 (16.5%)	
**Brain metastasis, %**						
Yes	6 (0.4%)	0 (0.0%)	1 (16.7%)	1 (16.7%)	4 (66.7%)	0.021
No/unknown	1633 (99.6%)	282 (17.3%)	533 (32.6%)	524 (32.1%)	294 (18.0%)	
**Liver metastasis, %**						
Yes	100 (6.1%)	8 (8.0%)	12 (12.0%)	4 (4.0%)	76 (76.0%)	< 0.001
No/unknown	1539 (93.9%)	274 (17.8%)	522 (33.9%)	521 (33.9%)	222 (14.4%)	
**Lung metastasis, %**						
Yes	29 (1.8%)	1 (3.4%)	3 (10.3%)	0 (0.0%)	25 (86.2%)	< 0.001
No/unknown	1610 (98.2%)	281 (17.5%)	531 (33.0%)	525 (32.6%)	273 (17.0%)	

Others^a^: American Indian/AK Native, Asian/Pacific Islander, unknown; Others^b^: Divorced, Separated, Widowed, unknown; SRCC: signet ring cell carcinoma; Others^c^: cystic, mucinous and serous neoplasms; SROC: surgery plus radiotherapy or chemotherapy; SCRT: surgery plus chemoradiotherapy; C16.0/C16.1: Cardia, NOS/Fundus of stomach; C16.2: Body of stomach; C16.3/C16.4: Gastric antrum/ Pylorus; C16.5/C16.6: Lesser curvature of stomach NOS/Greater curvature of stomach NOS; C16.8/C16.9: Overlapping lesion of stomach/ Stomach, NOS.

### Multiple logistic regression on the choice of treatment modality

The results of the multiple logistic regression analysis were presented in Table 2. There were no significant differences among treatment methods with respect to the age or marital status groups. Compared to male patients, females were more likely to receive surgery. Patients with C16.3/C16.4 gastric cancer were more inclined toward surgery compared to those with C16.0/C16.1 carcinoma. Additionally, patients with poorly differentiated or undifferentiated tumours tended to receive SCRT in comparison to nonsurgical treatment (OR = 2.228, 95% CI 1.550–3.204, *P* < 0.001). Patients with T2/T3/T4 or N2/N3 stage were more likely to choose SROC or SCRT than nonsurgical treatment, while patients with T3/T4 or N1/N2 stage were more likely to be treated without surgery compared to surgery only. Patients with larger tumours, distant metastasis, and the AJCC TNM stage IV were prone to receive nonsurgical treatment.

**Table 2:** Polytomous logistic regression for each treatment group (vs. no surgery) as the dependent variable of interest.

Variables	Surgery only versus no surgery			SROC versus no surgery			SCRT versus no surgery		
	OR	95% CI	*P*-value	OR	95% CI	*P*-value	OR	95% CI	*P*-value
**Age groups, years**									
≤ 40 years	Reference			Reference			Reference		
41–45 years	1.217	0.515–2.874	0.654	0.723	0.347–1.507	0.387	0.628	0.300–1.314	0.216
> 45 years	1.239	0.642–2.390	0.523	0.922	0.527–1.613	0.775	0.831	0.474–1.457	0.519
**Sex**									
Male	Reference			Reference			Reference		
Female	1.978	1.413–2.768	< 0.001	1.760	1.310–2.364	< 0.001	1.347	1.000–1.813	0.050
**Race**									
White	Reference			Reference			Reference		
Black	1.327	0.866–2.034	0.194	1.624	1.122–2.350	0.010	1.335	0.916–1.947	0.133
Others^a^	1.473	0.878–2.471	0.142	1.582	1.000–2.501	0.050	1.632	1.036–2.572	0.035
**Marital status**									
Married	Reference			Reference			Reference		
Unmarried	1.162	0.703–1.921	0.558	0.755	0.476–1.196	0.231	0.914	0.583–1.434	0.697
Others^b^	1.007	0.695–1.461	0.969	0.758	0.547–1.050	0.095	0.764	0.550–1.061	0.108
**Location**									
C16.0/C16.1	Reference			Reference			Reference		
C16.2	3.528	1.926–6.462	0.000	5.199	3.216–8.406	0.000	1.124	0.678–1.863	0.650
C16.3/C16.4	12.011	7.069–20.407	0.000	8.538	5.342–13.646	0.000	4.118	2.642–6.417	0.000
C16.5/C16.6	4.269	2.496–7.300	0.000	3.848	2.456–6.028	0.000	1.371	0.885–2.126	0.158
C16.7/C16.8	5.683	3.370–9.582	0.000	4.371	2.794–6.839	0.000	1.129	0.715–1.781	0.603
**Tumor size**									
≤ 3 cm	Reference			Reference			Reference		
3.1–5.0 cm	0.256	0.171–0.383	0.000	0.806	0.568–1.144	0.228	0.799	0.561–1.137	0.213
> 5.0 cm	0.254	0.165–0.392	0.000	0.699	0.480–1.016	0.061	0.808	0.556–1.174	0.263
**Grade**									
I/II	Reference			Reference			Reference		
III/IV	1.461	0.982–2.175	0.062	2.228	1.550–3.204	< 0.001	1.099	0.789–1.531	0.575
**Histological type**									
Adenocarcinoma	Reference			Reference			Reference		
SRCC	1.982	1.392–2.823	< 0.001	1.802	1.318–2.464	< 0.001	1.314	0.955–1.808	0.093
Others^c^	4.016	0.800–20.159	0.091	2.038	0.407–10.189	0.386	3.747	0.831–16.901	0.086
**AJCC TNM stage**									
I	Reference			Reference			Reference		
II	0.091	0.047–0.176	< 0.001	0.840	0.442–1.596	0.595	2.237	1.136–4.402	0.020
III	0.143	0.072–0.283	< 0.001	1.750	0.900–3.404	0.099	3.905	1.935–7.883	< 0.001
IV	0.015	0.008–0.028	< 0.001	0.147	0.081–0.268	< 0.001	0.070	0.034–0.143	< 0.001
**AJCC_T stage**									
T1	Reference			Reference			Reference		
T2	0.717	0.379–1.356	0.306	5.889	3.146–11.026	< 0.001	6.608	3.454–12.642	< 0.001
T3	0.214	0.136–0.335	< 0.001	3.432	2.196–5.365	< 0.001	6.117	3.846–9.727	< 0.001
T4	0.324	0.212–0.494	< 0.001	4.272	2.738–6.665	< 0.001	3.429	2.126–5.530	< 0.001
**AJCC_N stage**									
N0	Reference			Reference			Reference		
N1	0.087	0.052–0.145	< 0.001	0.766	0.540–1.088	0.137	0.919	0.643–1.312	0.641
N2	0.451	0.255–0.799	0.006	2.768	1.728–4.435	< 0.001	4.018	2.513–6.423	< 0.001
N3	1.248	0.707–2.201	0.445	6.341	3.759–10.697	< 0.001	6.395	3.76–10.875	< 0.001
**AJCC_M stage**									
M0	Reference			Reference			Reference		
M1	0.056	0.036–0.088	< 0.001	0.126	0.091–0.174	< 0.001	0.027	0.017–0.043	< 0.001

Others^a^: American Indian/AK Native, Asian/Pacific Islander, unknown; Others^b^: Divorced, Separated, Widowed, unknown; SRCC: signet ring cell carcinoma; Others^c^: cystic, mucinous and serous neoplasms; SROC: surgery plus radiotherapy or chemotherapy; SCRT: surgery plus chemoradiotherapy; OR: odds ratio; CI: confidence Interval; C16.0/C16.1: Cardia, NOS/Fundus of stomach; C16.2: Body of stomach; C16.3/C16.4: Gastric antrum/ Pylorus; C16.5/C16.6: Lesser curvature of stomach NOS/Greater curvature of stomach NOS; C16.8/C16.9: Overlapping lesion of stomach/Stomach, NOS.

### Survival outcomes of different treatments for EOGC patients

**Figure 2:**
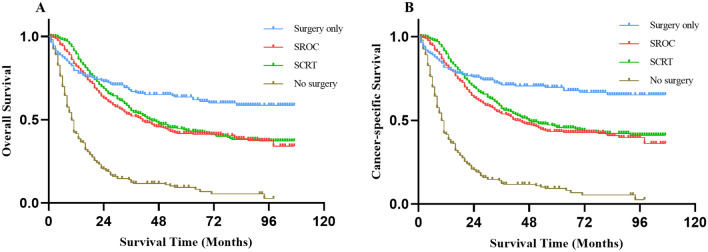
Kaplan–Meier survival curves comparing each treatment for EOGC patients. SROC, surgery plus radiotherapy or chemotherapy; SCRT, surgery plus chemoradiotherapy.

In analyses of the entire cohort, patients treated with surgery alone had superior survival than those treated with nonsurgical treatment [1 year OS rate (79.50% vs. 40.40%, *P* < 0.001), 3-year OS rate (66.90% vs. 11.20%, *P* < 0.001), 5 year OS rate (63.70% vs. 8.00%, *P* < 0.001)]. In addition, there was no significant difference between the survival of SROC and SCRT (median OS: 41 months vs. 45 months, *P* = 0.184). The 1-year OS rate of patients undergoing surgery alone were lower than those undergoing SROC and SCRT (79.50% vs. 82.10% vs. 89.50%, *P* = 0.001) while the 3-year OS rate (66.90% vs. 52.20% vs. 55.40%, *P* = 0.001) and 5-year OS rate (63.70% vs. 41.70% vs. 43.30%, *P* = 0.001) were reversed. The same was true for the CSS. The results of survival analysis across different treatment categories are displayed in Fig. 2 as survival curves and Table S1 lists the *P* values for paired comparisons of therapeutic methods.

### Subgroup analysis based on histological type, TNM stage and tumour size

**Figure 3:**
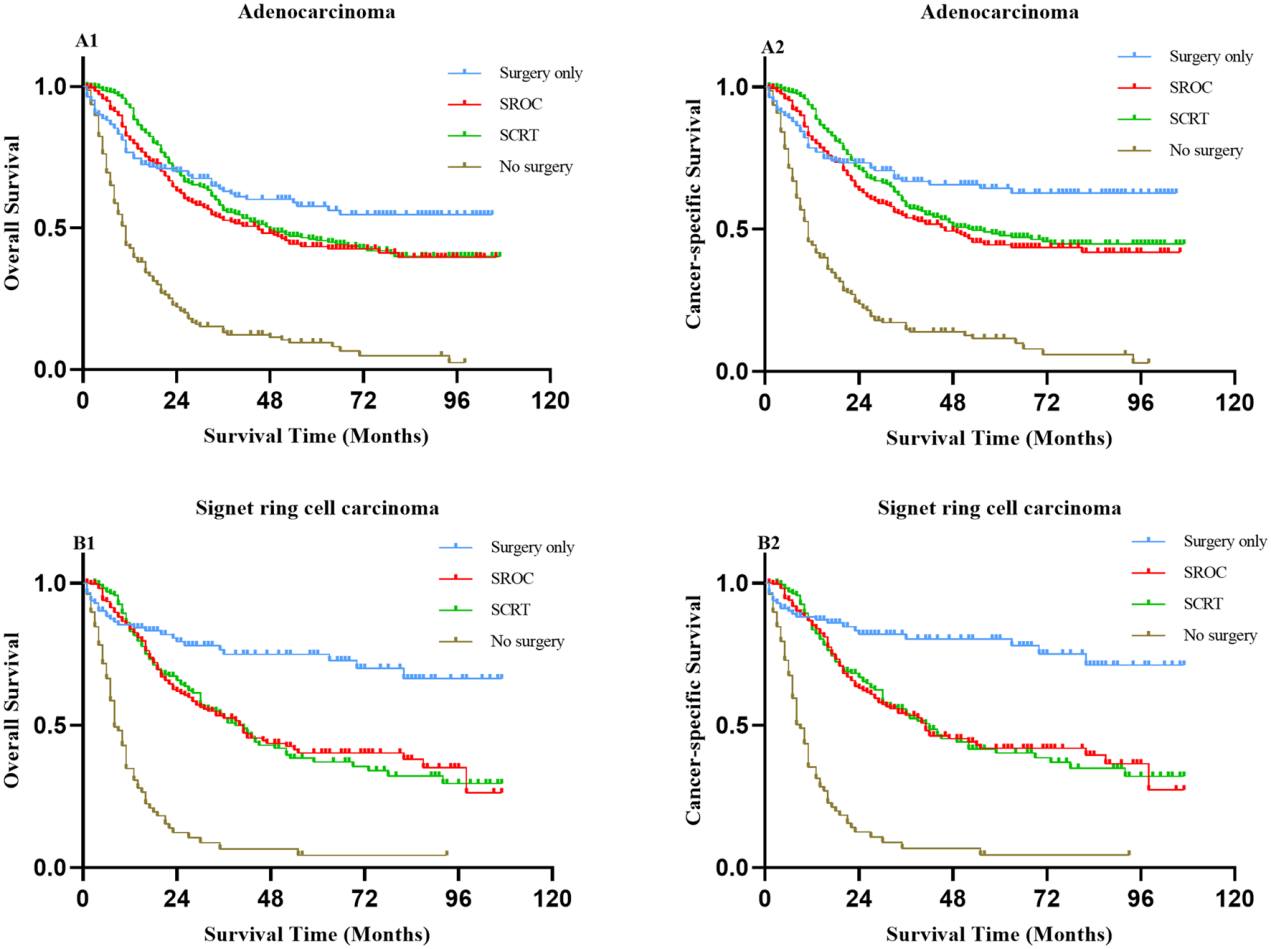
Kaplan–Meier survival curves for overall survival (**A1**, **B1**) and cancer-specific survival (**A2**, **B2**) in patients with gastric adenocarcinoma and SRCC undergoing different treatments respectively. SROC, surgery plus radiotherapy or chemotherapy; SCRT, surgery plus chemoradiotherapy; SRCC, signet ring cell carcinoma.

**Figure 4:**
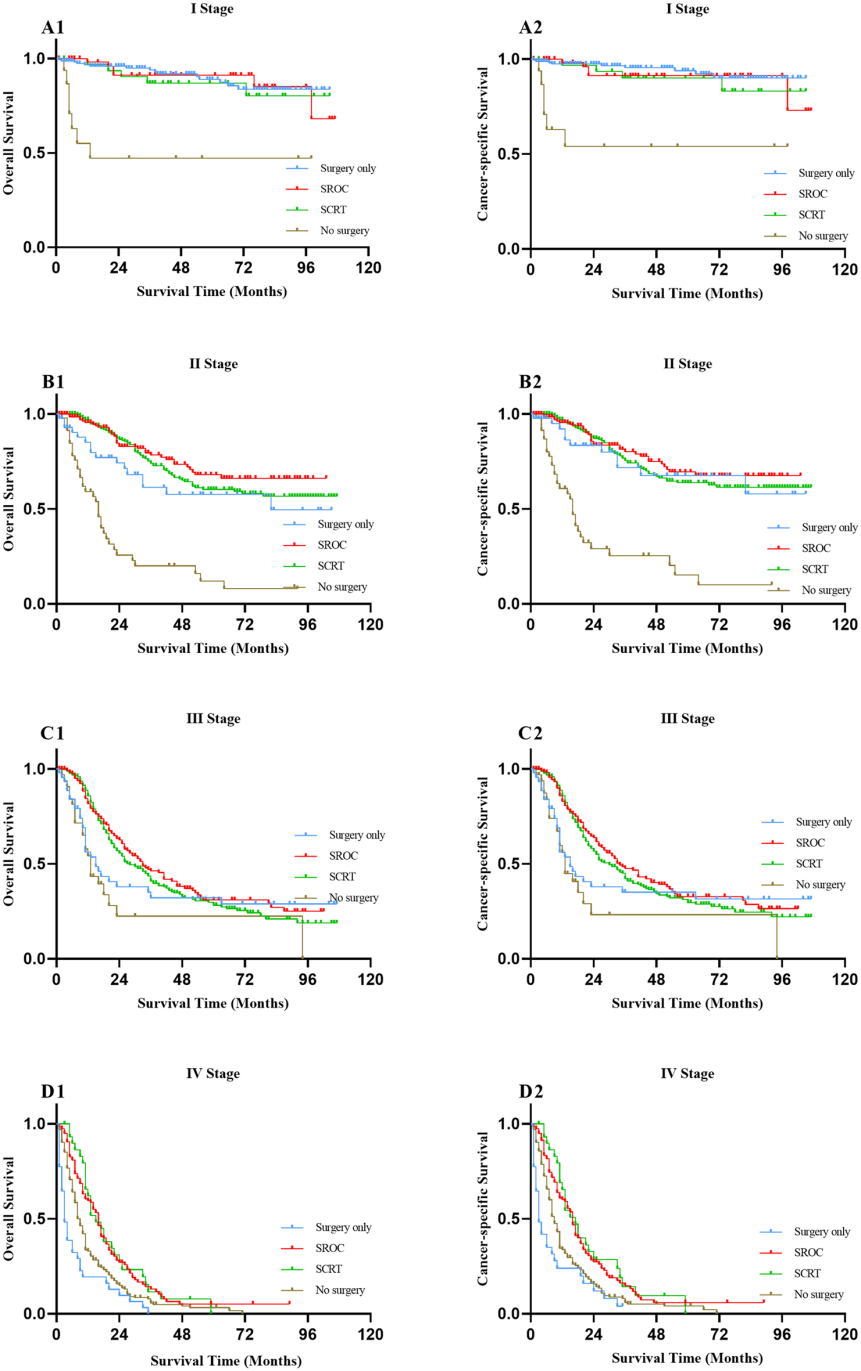
Kaplan–Meier survival curves for overall survival (**A1**, **B1**, **C1**, **D1**) and cancer-specific survival (**A2**, **B2**, **C2**, **D2**) in patients with TNM stage I, II, III, IV undergoing different treatments respectively. SROC, surgery plus radiotherapy or chemotherapy; SCRT, surgery plus chemoradiotherapy.

**Figure 5:**
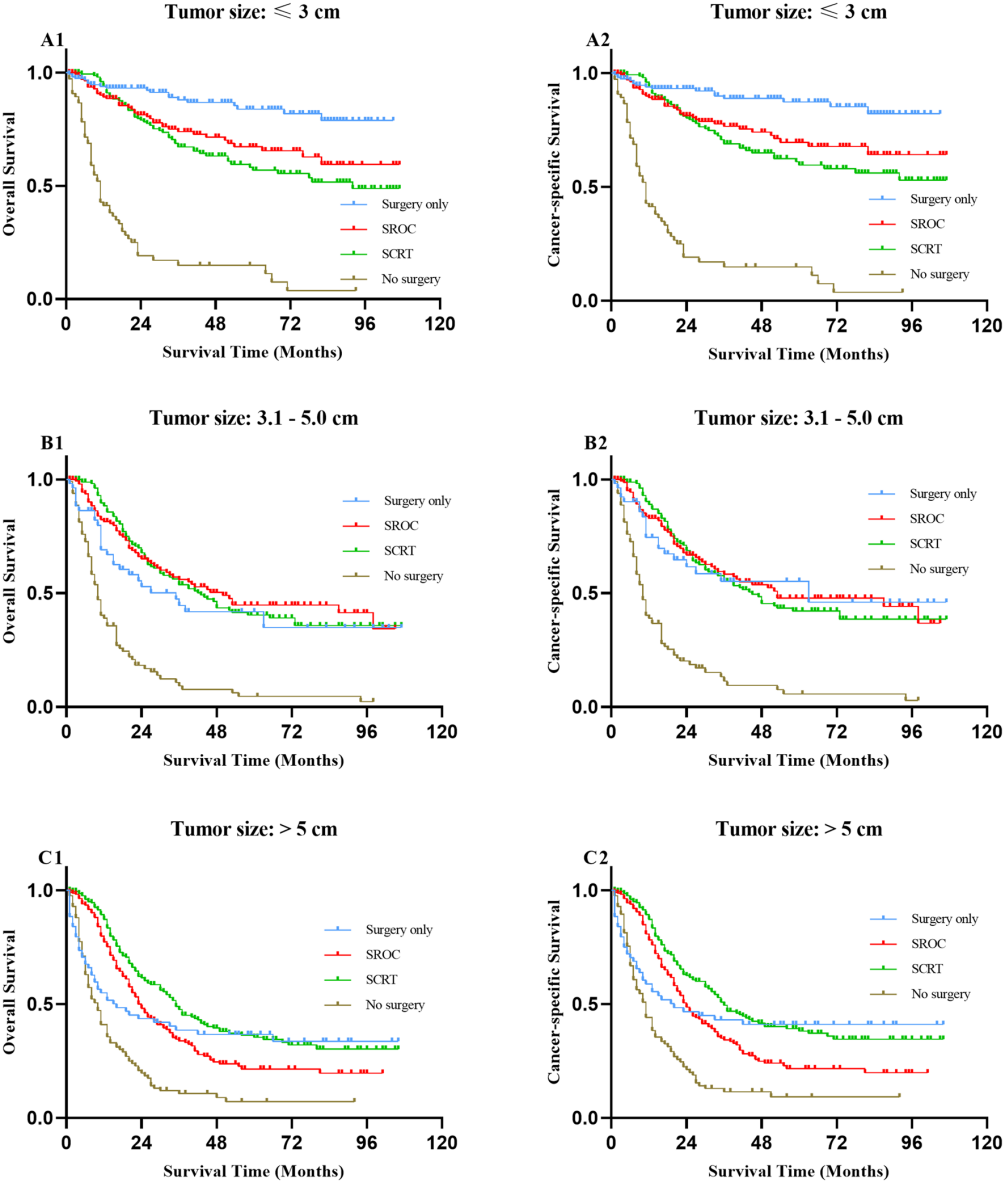
Kaplan–Meier survival curves for (**A1**, **B1**, **C1**) overall survival and (**A2**, **B2**, **C2**) cancer-specific survival in patients with tumor size ≤ 3 cm, 3.1–5 cm, > 5 cm undergoing different treatments respectively. SROC, surgery plus radiotherapy or chemotherapy; SCRT, surgery plus chemoradiotherapy.

Since treatment strategies might be affected by the histological type, TNM stage and tumour size, we further conducted subgroup analyses on the basis of these factors. When the histological type was SRCC, the OS and CSS of surgery alone were better than those of SROC and SRCT, but there was no significant difference in adenocarcinoma (Fig. 3 and Table S2). When subjects were categorized into subgroups according to TNM stage, no differences were found between SROC and SCRT for patients in all stages with regard to OS and CSS (all *P* > 0.05). There was no significant difference in the survival of patients undergoing surgery alone compared with SROC and SCRT for different TNM stage categories (all *P* > 0.05) except for stage IV (Fig. 4 and Table S3). In the subgroup of tumour size ≤ 3 cm, patients undergoing surgery alone had better 5-year OS and CSS rates than those with SROC [5 year OS (83.80% vs. 67.20%, *P* = 0.002, 5 year CSS (87.30% vs. 69.60%, *P* = 0.002)] and SCRT [5 year OS (83.80% vs. 56.90%, *P* < 0.001, 5 year CSS (87.30% vs. 59.70%, *P* < 0.001)], while there were no significant differences in 5-year OS or CSS rates between SROC and SCRT (*P* > 0.05). However, there was similar survival among the patients who underwent surgery alone or SROC or SCRT when the tumour size was between 3.1 and 5 cm. Of note, patients who underwent SCRT had a higher 5-year OS (35.40% vs. 21.50%, *P* = 0.002) rate and 5-year CSS rate (38.20% vs. 21.70%, *P* = 0.001) than those treated by SROC in the tumour size > 5 cm subgroup (Fig. 5 and Table S4). In addition, regardless of histological type, TNM stage and tumour size, patients treated conservatively had poorer OS and CSS than those who received the other three treatments (all *P* < 0.05).

### Stratified Cox regression risk analysis for each treatment

**Figure 6:**
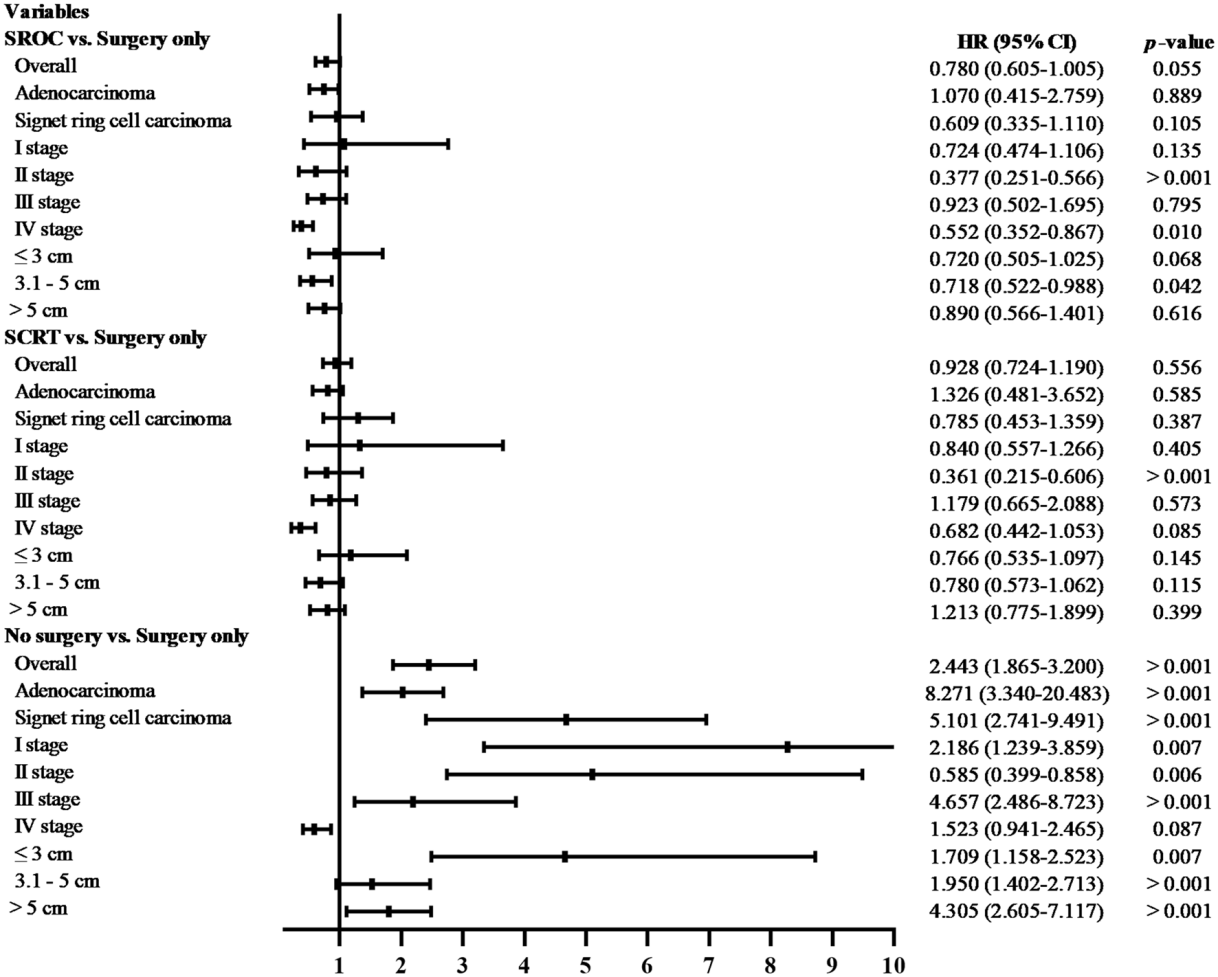
Overall survival of study subgroups in multivariable analyses. (Surgery alone as a reference). SROC, surgery plus radiotherapy or chemotherapy; SCRT, surgery plus chemoradiotherapy; HR, hazard ratio; CI, confidence Interval.

The stratified HRs further revealed that overall survival was significantly better among patients undergoing surgery than those receiving chemotherapy alone, radiotherapy alone, or chemoradiotherapy in the stage I, II, and III subgroups, while there were no significant differences in prognosis between patients treated with surgery alone and those treated with SROC and SCRT (both *P* > 0.05). In addition, compared with patients who received surgery alone, conservative treatment (HR = 0.585, 95% CI 0.399–0.858, *P* = 0.006), SROC (HR = 0.377, 95% CI 0.251–0.566, *P* < 0.001) and SCRT (HR = 0.361; 95% CI 0.215–0.606; *P* < 0.001) in patients had a lower risk of overall death at stage IV. Most notably, patients who received nonsurgical treatment had the highest risk of overall death, which was more prominent among patients with a tumour size ≤ 3 cm (HR = 4.657, 95% CI 2.486–8.723, *P* < 0.001) than among those with a tumour size > 5 cm (HR = 1.709, 95% CI 1.158–2.523, *P* = 0.007) (Fig. 6). Regardless of histological type, the risk of death from surgery alone was not significantly different from that of SROC and SRCT. The same was true for the risk of cancer-specific death. (Fig. S1).

### Cause of death

**Figure 7:**
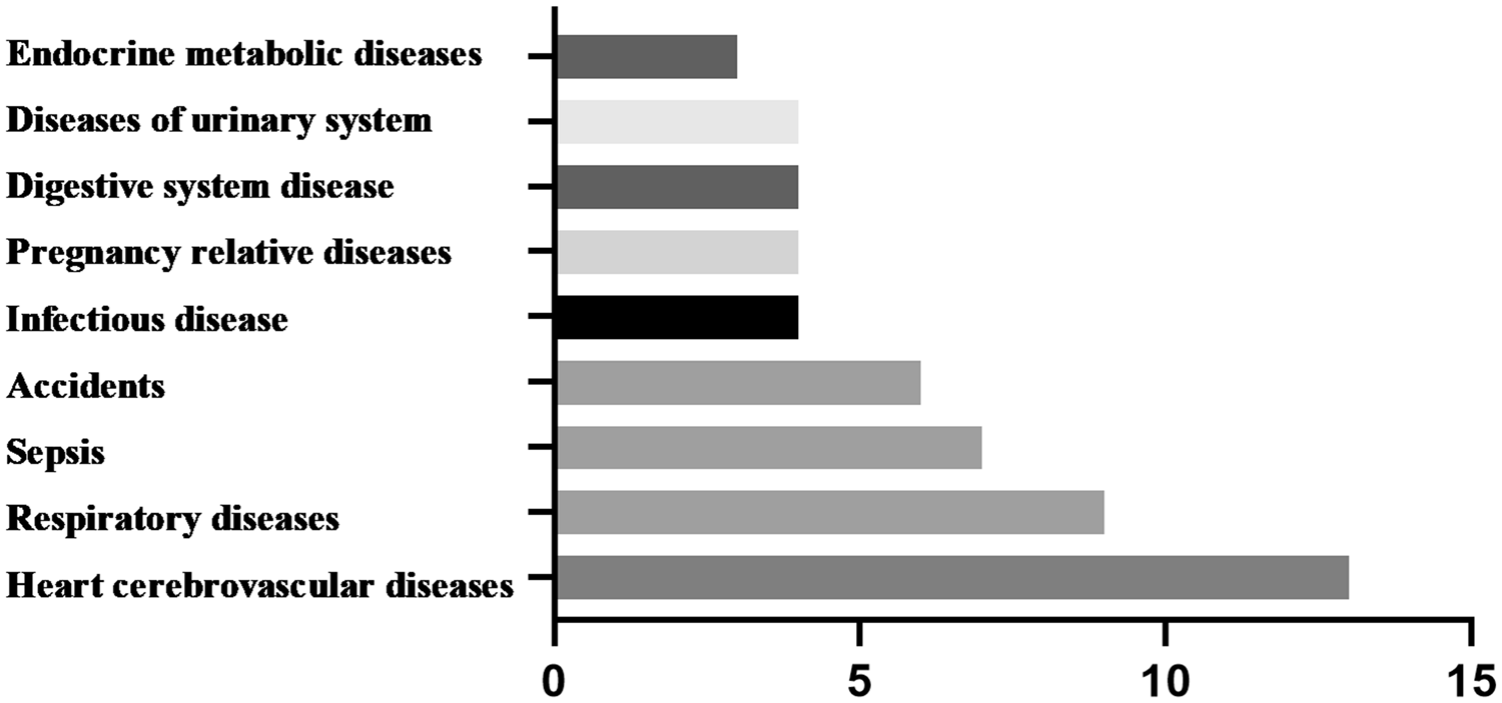
The main causes of non-tumor death.

The mortality rate of EOGC patients was 50.82%, most of which was attributed to the tumour itself, accounting for 93.52%. The main causes of nontumor death were heart cerebrovascular diseases (24.07%), followed by respiratory diseases (16.67%), sepsis (12.96%), accidents (11.11%), and others (35.19%). (Fig. 7).

## Discussion

Due to the low incidence of EOGC, few clinical studies have been published concerning its treatment strategy and survival. The published literature did not identify any differences in the treatment methods of EOGC and ordinary GC, whereas there were significant differences in survival. The current approach is still mainly surgical treatment, supplemented by chemotherapy, radiotherapy, targeted therapy, or immunotherapy^24^. Perioperative chemotherapy and postoperative chemoradiation are the preferred approaches for patients with localized resectable disease and patients undergoing less than a D2 lymph node dissection, respectively^25,26^. Nevertheless, whether these treatments are also suitable for young patients remains unclear. Consequently, this study explored the effects of different treatment modalities on the prognosis of EOGC patients in the SEER database.

In certain retrospective studies, the proportion of EOGC patients in women was higher than that in men, which may be associated with high percentages of oestrogen receptor positivity in young female patients^22,27^. However, some studies have suggested that male patients with EOGC are more common than female patients^28,29^. Similar results were found in our study, which was dominated by male EOGC patients, who were prone to undergo nonsurgical treatment compared to male patients. Additionally, there was a high incidence of stage T3/T4 lesions and low differentiation among these EOGC patients, which was consistent with the results of previous studies^30,31^. Our logistic regression analysis suggested that patients in the M1 stage preferred to receive nonsurgical treatment in comparison with patients in the M0 stage. The reasons for the above may be related to the biological characteristics of GC, and the delayed diagnosis of GC in young patients on account of the lack of early symptoms in early-stage^32,33^.

In the present study, we observed that the majority of young patients received active surgical treatment. Young patients who received surgery alone had a better 3/5 year OS and CSS rate than those who received surgery plus chemotherapy, radiotherapy or chemoradiotherapy but had a poorer 1 year OS and CSS rate. Interestingly, there were no differences in the risk of death after adjusting for these potential confounding factors. Of note, patients with EOGC who received surgery plus chemotherapy, radiotherapy or chemoradiotherapy did not receive extra survival benefits. Our finding appears to be inconsistent with the recommendations of management guidelines for GC^34^. The current NCCN guidelines indicate that perioperative chemotherapy is the preferred treatment for resectable T2 tumours or higher and any N tumours lesions while surgery alone should be considered by patients with T1b tumours. In addition, for patients with unresectable and/or metastatic diseases, the best supportive care and palliative treatment can be provided according to the patient's functional status and previous treatment^34^. The reasons for this observation are undoubtedly complex and multifactorial, but the main reason may be that EOGC cannot not the tolerate toxicity or adverse reactions of chemoradiotherapy. Moreover, multiple studies have shown that not every stage II or III GC patient necessarily benefits from perioperative chemotherapy and can even result in adverse events^35–37^. Collectively, additional chemoradiotherapy may not be suitable for all young patients with resectable GC. In addition, several studies have suggested that patients with MSI-high cancers may have an adverse oncologic outcome when treated with surgery plus perioperative or adjuvant chemotherapy while having improved survival with surgery alone^38–40^. Unfortunately, lack of MSI consistently occurs in EOGC patients^41,42^. Most importantly, our study could not provide the expression of MSI in research objects. To date, reports on chemoradiotherapy in young GC patients have been limited to a few small retrospective studies. Thus, well-designed prospective clinical studies of high quality in the young population are required to corroborate our findings.

Our further subgroup analysis of tumour histological type, TNM staging and size stratification yielded some different outcomes. In our study, patients with SRCC were more likely to choose surgery alone or SROC than patients with gastric adenocarcinoma. Stratified analysis showed that the survival of surgery alone in patients with SRCC was better than SROC and SCRT, but there was no significant difference in gastric adenocarcinoma patients, which was consistent with the previous study that SRCC was not sensitive to radiotherapy and chemotherapy. A large retrospective study involving 1520 patients undergoing radical gastrectomy showed that the survival rate of patients with SRCC was higher than that of patients with gastric adenocarcinoma^43^. Another study included 218 SRCC patients and 1221 non SRCC patients showed that there was a difference in the overall 5 year survival rate between SRCC and non SRCC patients (44.9% vs. 36.0%, *P* = 0.013)^44^. Our study also obtained the above similar results. In the present study, a large proportion of patients with stage I–III disease received SROC or SCRT. Notably, there were no significant differences in OS or CSS among those stage I-III EOGC patients who received surgery alone compared to SROC or SCRT. These therapeutics may indicate overtreatment because chemoradiotherapy for stage I patients is not explicitly recommended in previous and current guidelines, while the preferred treatment for stage II or III patients is inconsistent with the guidelines^34,35,45^. Similar survival results were noted in subgroup analyses of patients with tumours sizes of 3.1–5 cm. Limiting the analysis to patients with tumour size ≤ 3 cm, the survival was superior in the surgery alone group at 1, 3 and 5 years of follow-up in comparison to the other three treatment groups. Moreover, the survival of patients undergoing SROC did not differ from those receiving SCRT regardless of tumour TNM stage or tumour ≤ 5 cm. EOGC patients undergoing nonsurgical treatment had the worst survival in all tumour sizes and TNM stage I–III subgroups but had relatively good survival in stage IV. In addition, we did not observe a significant difference among the three surgical groups in the risk of overall death or cancer-specific death of patients with stage I–III tumours in the Cox proportional hazards regression models. Similar results were seen when modelling was based on different tumour size subgroups. Significantly, the risk of death was higher in all subgroups of patients with nonsurgical treatment than in those treated surgically, except for the stage IV subgroup of patients [overall death (HR: 0.585, 95% CI 0.399–0.858, *P* = 0.006), cancer-specific death (HR: 0.628, 95% CI 0.421–0.938, *P* = 0.023)]. All of the above findings indicate that the tumour TNM stage plays a crucial role in treatment decision-making. Nevertheless, the choice of treatment mainly depends on patient performance status and medical comorbidities as well as the toxicity profile of the regimen^46^. In addition, whether to carry out radiotherapy or chemotherapy is also connected to the patient's economic situation and cultural customs^47,48^. Hence, our above results may have great clinical and economic application value. An unreasonable increase in the use of radiotherapy or chemotherapy will fail to benefit young patients and can also harm them and increase the social and economic burden. Consequently, the rational application of radiotherapy and chemotherapy in the treatment of resectable EOGC patients should be discussed and evaluated in further research.

Inevitably, some limitations should be considered in our present study. First, this was a retrospective study based on the SEER database, which inevitably had intrinsic selection bias in therapeutic strategies, such as the analysis of promiscuous survival due to nonrandomized treatment assignments. Second, some significant factors affecting the prognosis of EOGC, such as infection with *Helicobacter pylori* bacteria, dietary habits, general health status, and laboratory and imaging examinations, could be unavailable in the SEER registry, which may have contributed to our findings. It is critical to consider the above factors for the selection of treatment strategies for EOGC patients. Third, there was a lack of further details of treatment, such as the order of the therapy, resection margins, the timing of surgery, the specific chemotherapy and radiation contents, treatment toxicity, and treatment willingness of patients, which should be determined in future research. In addition, our study was conducted in a small number of patients, which could have influenced our observations. Based on these problems, we attempted to reduce potential bias by adjusting the HR for the influence of each treatment method on survival. Therefore, the results of the present study could be useful for clinicians to select appropriate treatment decisions and develop appropriate follow-up strategies. There is an urgent need for prospective multicentre collaborative studies with larger samples to select optimal treatment strategies to improve survival and quality of life for GOGC patients and to obtain high-quality evidence.

In summary, the results of our study demonstrated similar long-term survival outcomes among surgery alone, SROC and SCRT for EOGC patients after adjusting for potential confounding factors, which indicated that additional radiotherapy, chemotherapy or chemoradiotherapy does not bring coordinated survival benefits. More efforts with prospective multicentre collaborative trials that evaluate the appropriate treatment for EOGC patients are still needed to obtain high-quality evidence.

## Supplementary Information

Supplementary Information.

## Data Availability

Publicly available datasets were analyzed in this study. This data can be found in the Surveillance, Epidemiology, and End Results (SEER) database (https://seer.cancer.gov/).
