# Supplementary material for: Comparison of treatment strategies and survival of early-onset gastric cancer: a population-based study
Source: Sci Rep. 2022 Apr 15;12:6288. doi: 10.1038/s41598-022-10156-5 (PMC9012810; doi:10.1038/s41598-022-10156-5)
Supplement: Supplementary file 1 — Supplementary Information. [file 41598_2022_10156_MOESM1_ESM.pdf]

Table S1 *P*-values for paired comparison of therapeutic methods in OS and CSS.

Treatment	Surgery only		SROC		SCRT		No surgery	
	OS	CSS	OS	CSS	OS	CSS	OS	CSS
**Surgery only**	—	—	0.001	< 0.001	0.004	0.001	< 0.001	< 0.001
**SROC**	0.001	< 0.001	—	—	0.184	0.13	< 0.001	< 0.001
**SCRT**	0.004	0.001	0.184	0.13	—	—	< 0.001	< 0.001
**No surgery**	< 0.001	< 0.001	< 0.001	< 0.001	< 0.001	< 0.001	—	—

SROC, surgery plus radiotherapy or chemotherapy; SCRT, surgery plus chemoradiotherapy; OS, overall survival; CSS, cancer-specific survival.

Table S2 *P*-values for paired comparison of therapeutic methods for OS and CSS according to tumour histological type.

Size	Treatment	Surgery only		SROC		SCRT		No surgery	
		OS	CSS	OS	CSS	OS	CSS	OS	CSS
**Adenocarcinoma**	**Surgery only**	—	—	0.110	0.019	0.343	0.137	< 0.001	< 0.001
	**SROC**	0.110	0.019	—	—	0.199	0.115	< 0.001	< 0.001
	**SCRT**	0.343	0.137	0.199	0.115	—	—	< 0.001	< 0.001
	**No surgery**	< 0.001	< 0.001	< 0.001	< 0.001	< 0.001	< 0.001	—	—
**Signet ring cell carcinoma**	**Surgery only**	—	—	< 0.001	< 0.001	< 0.001	< 0.001	< 0.001	< 0.001
	**SROC**	< 0.001	< 0.001	—	—	0.924	0.986	< 0.001	< 0.001
	**SCRT**	< 0.001	< 0.001	0.924	0.986	—	—	< 0.001	< 0.001
	**No surgery**	< 0.001	< 0.001	< 0.001	< 0.001	< 0.001	< 0.001	—	—

Table S3 *P*-values for paired comparison of therapeutic methods for OS and CSS according to tumour TNM stage.

AJCC_TNM	Treatment	Surgery only		SROC		SCRT		No surgery	
		OS	CSS	OS	CSS	OS	CSS	OS	CSS
**I Stage**	**Surgery only**	—	—	0.931	0.517	0.58	0.342	< 0.001	< 0.001
	**SROC**	0.931	0.517	—	—	0.736	0.816	< 0.001	< 0.001
	**SCRT**	0.58	0.342	0.736	0.816	—	—	0.002	0.003
	**No surgery**	< 0.001	< 0.001	< 0.001	< 0.001	0.002	0.003	—	—
**II Stage**	**Surgery only**	—	—	0.056	0.453	0.206	0.784	< 0.001	< 0.001
	**SROC**	0.056	0.453	—	—	0.291	0.417	< 0.001	< 0.001
	**SCRT**	0.206	0.784	0.291	0.417	—	—	< 0.001	< 0.001
	**No surgery**	< 0.001	< 0.001	< 0.001	< 0.001	< 0.001	< 0.001	—	—
**III Stage**	**Surgery only**	—	—	0.09	0.084	0.24	0.203	0.292	0.36
	**SROC**	0.09	0.084	—	—	0.333	0.338	< 0.001	< 0.001
	**SCRT**	0.24	0.203	0.333	0.338	—	—	< 0.001	< 0.001
	**No surgery**	0.292	0.36	< 0.001	< 0.001	< 0.001	< 0.001	—	—

IV Stage	**Surgery only**	—	—	< 0.001	< 0.001	< 0.001	0.001	0.004	0.018
	**SROC**	< 0.001	< 0.001	—	—	0.767	0.627	< 0.001	< 0.001
	**SCRT**	< 0.001	0.001	0.767	0.627	—	—	0.017	0.012
	**No surgery**	0.004	0.018	< 0.001	< 0.001	0.017	0.012	—	—

SROC, surgery plus radiotherapy or chemotherapy; SCRT, surgery plus chemoradiotherapy; OS, overall survival; CSS, cancer-specific survival.

Table S4 *P*-values for paired comparison of therapeutic methods for OS and CSS according to tumour size.

**Size**	**Treatment**	**Surgery only**		**SROC**		**SCRT**		**No surgery**	
		**OS**	**CSS**	**OS**	**CSS**	**OS**	**CSS**	**OS**	**CSS**
**≤ 3 cm**	**Surgery only**	—	—	0.002	0.002	0	0	< 0.001	< 0.001
	**SROC**	0.002	0.002	—	—	0.294	0.289	< 0.001	< 0.001
	**SCRT**	< 0.001	0	0.294	0.289	—	—	< 0.001	< 0.001
	**No surgery**	< 0.001	< 0.001	< 0.001	< 0.001	< 0.001	< 0.001	—	—
**3.1–5 cm**	**Surgery only**	—	—	0.222	0.809	0.209	0.853	< 0.001	< 0.001
	**SROC**	0.222	0.809	—	—	0.874	0.792	< 0.001	< 0.001
	**SCRT**	0.209	0.853	0.874	0.792	—	—	< 0.001	< 0.001
	**No surgery**	< 0.001	< 0.001	< 0.001	< 0.001	< 0.001	< 0.001	—	—
**> 5 cm**	**Surgery only**	—	—	0.842	0.683	0.048	0.113	0.001	0.001
	**SROC**	0.842	0.683	—	—	0.002	0.001	< 0.001	< 0.001
	**SCRT**	0.048	0.113	0.002	0.001	—	—	< 0.001	< 0.001
	**No surgery**	0.001	0.001	< 0.001	< 0.001	< 0.001	< 0.001	—	—

SROC, surgery plus radiotherapy or chemotherapy; SCRT, surgery plus chemoradiotherapy; OS, overall survival; CSS, cancer-specific survival.

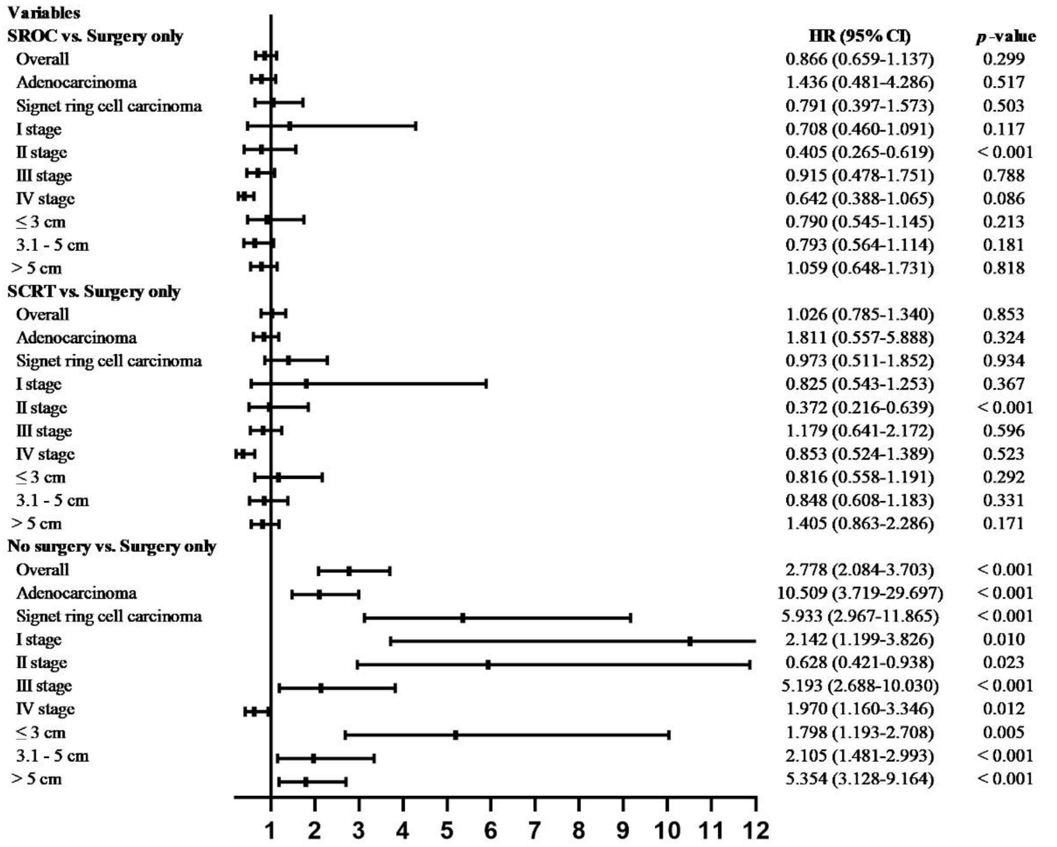

Figure S1 Cancer-specific survival of study subgroups in multivariable analyses. (Surgery alone as a reference)

SROC: surgery plus radiotherapy or chemotherapy; SCRT: surgery plus chemoradiotherapy; HR: hazard ratio; CI: confidence Interval;
